# News and Coming Events

**Published:** 1909-09-18

**Authors:** 


### News and Coming Events.

Among the latest contributions to King Edward's Hospital Fund for London is the sum of £105, being the annual subscription of his Majesty the King, founder and patron of the fund.

The National Association for Promoting the Welfare of the Feeble-minded has convened a Conference of After-care Committees to be held at Leicester on Thursday, October 28. Members of boards of guardians are being asked to send representatives.

Sir Edgar Speyer will take the chair at a festival banquet at the Whitehall Rooms, Hôtel Métropole, on Wednesday, November 24, for the purpose of raising the sum of £12,000 in aid of the Great Northern Central Hospital, which, unless immediate help is forthcoming, will have to close at least 50 beds by the end of the year.

The distribution of prizes and an address to the students of Charing Cross Hospital Medical School will take place in the out-patients' hall at the hospital (entrance in King William Street), on Monday, October 4, 1909, at 4 o'clock, by the Right Hon. the Viscount Ridley (Chairman of the hospital). Tea and coffee will be served at the conclusion of the proceedings.

Lord Tredegar last week opened extensions, costing £10,000, to the Rest, Porthcawl, a convalescent home. Lord Tredegar said that in the days when the institution was founded, half a century ago, the word rest was quite a new one. Now rest was the medical way of dealing with all ailments when they did not know what else to do. The nation had realised the importance of trying to make children healthy before teaching them the ABC.

The Anglo-American Oil Company's steamer *Cheyenne*, which put into Falmouth the week before last with suspected cases of cholera on board, left for America last week after having been in quarantine for four days. It is stated that the men in question were not suffering from cholera, but from severe dyspepsia after indiscretions in diet. The port sanitary medical officer granted the *Cheyenne* a clean bill of health at the request of the American Consul at Plymouth.

The date of the third International Congress of School Hygiene, to be held in Paris next year, has been postponed, so we are officially informed, from the end of March, 1910, to the first week of August, 1910. The Congress and Associated Exhibition will accordingly be open from August 2 until August 7 of next year. The French Organising Committee state that this postponement has been asked for from several quarters, and is generally considered advisable.

The Baroness von Eckardstein has consented to open the sale of patients' work at the British Home and Hospital for Incurables, Streatham, on Wednesday, Novermber 24. The money raised at this sale does not go to the general funds, but is distributed amongst the patients, and thus provides them with a little pocket money for the year. Articles for the sale for the benefit of those patients who are too afflicted and crippled to work for themselves, and donations will be acknowledged by the Secretary, Mr. Edgar Penman, 72 Cheapside, E.C.

The autumn term of the School of Massage and Electrical Treatment, recently instituted at the Hospital for Epilepsy and Paralysis, etc., Maida Vale, commences on September 20. Applications for admission should be sent to the Secretary of the hospital without delay.

An outbreak of beri-beri is announced to have occurred on board the Brazilian cruiser *Carroza*, recently arrived in the Tyne with men for the new battleship being built there for the Brazilian Government. It was found necessary for the Tyne Port Sanitary Authority to remove five men to the floating hospital suffering from the disease, and one of them has died.

St. Mary's Hospital Medical School, Paddington, W.—The opening of the Winter Session will take place on Friday, October 1, when the prizes and awards for the sessions 1908-9 will be presented by Dr. H. A. Miers, F.R.S., Principal of the University of London, at three o'clock. The annual dinner of past and present students will be held at the Café Royal, Regent Street, at seven o'clock the same evening.

According to the St. Petersburg correspondent of the *Times*, the City Councillors of that city have resolved upon the immediate construction of additional filters on the plans and terms offered by the firm of Siemens and Halske. The only other competitor was a Russian firm. Cholera last week continued to claim there an average of 25 victims daily. The total for the year is: 5,769 deaths, of which 88 per cent. are among the working classes. Preventive inoculation was applied to 53,162 inhabitants, of whom only 12 contracted the disease, and eight of these recovered.

The official opening of the 75th winter session of the Medical School of the Middlesex Hospital will take place at the hospital on Friday, October 1, at 3 p.m. The introductory address will be delivered by Dr. J. Strickland Goodall, the Lecturer on Physiology, after which the prizes gained during the previous year will be distributed by Mr. E. H. Shackleton, C.V.O. Admission to the opening ceremony will be by card only, and applications should be made to the Secretary-Superintendent, Mr. F. Clare Melhado, as early as possible, as the seating space will be rather limited. In the evening the customary banquet of past and present students of the Medical School will be held at the Trocadero Restaurant.

